# Nutritional improvement following growing rod surgery in children with early onset scoliosis

**DOI:** 10.1007/s11832-014-0586-z

**Published:** 2014-04-24

**Authors:** Karen S. Myung, David L. Skaggs, George H. Thompson, John B. Emans, Behrooz A. Akbarnia, Growing Spine Study Group

**Affiliations:** 1 Children’s Hospital Los Angeles, 4650 Sunset Blvd., MS #69, Los Angeles, CA 90027 USA; 2 Rainbow Babies and Children’s Hospital, Case Western Reserve University, Cleveland, OH USA; 3 Department of Orthopaedic Surgery, Children’s Hospital and Harvard Medical School, Boston, MA USA; 4 San Diego Center for Spinal Disorders, La Jolla, CA USA; 5 Department of Orthopedic Surgery, University of California-San Diego, San Diego, CA USA; 6 Growing Spine Foundation, Milwaukee, WI USA

**Keywords:** Growing rod technique, Early onset scoliosis, Nutrition, Pulmonary

## Abstract

**Purpose:**

We aimed to evaluate the nutritional status of children with early onset scoliosis (EOS) and to determine if treatment with growing rod instrumentation improves weight percentile.

**Methods:**

Data was retrospectively collected on 88 EOS patients treated with growing rods at six institutions. Mean age at surgery was 5.8 years, and mean Cobb angle was 75°. All patients were followed for at least 2 years (mean 4 years). Weights were converted to normative percentiles based on the patients’ age and gender.

**Results:**

Preoperatively, 47 % (41/88) of patients were <5 percentile for weight, thus failing to thrive. There was a significant increase in mean postoperative weight percentiles at latest follow-up (*p* = 0.004). 49 % of patients gained weight, with a mean of 18 percentile. A significant relationship exists between age at initial surgery and percentile weight gain (*p* < 0.005), with children <4 years old not demonstrating postoperative improvement. This relationship was not confounded by preoperative weight, preoperative Cobb angle, or years of follow-up (*p* > 0.05). Children with neuromuscular and syndromic diagnoses do not appear to improve their mean nutritional status after surgery when compared to patients with idiopathic or congenital/structural scoliosis (*p* = 0.006).

**Conclusion:**

Following growing rod treatment, there was significant improvement in nutritional status in approximately 50 % of patients, similar to that reported with VEPTR. Neuromuscular and syndromic patients did not experience nutritional improvement post-operatively. These findings support the theory that growing rods improve the clinical status of EOS patients, as nutritional improvement is one outcome of improved clinical status. The relationship between age at initial surgery and nutritional improvement is intriguing.

## Introduction

Children with early-onset scoliosis (EOS) may have complex chest wall and/or spine deformities that compromise pulmonary function and ultimately contribute to thoracic insufficiency syndrome (TIS) [1]. In these children, the energy expenditure dedicated to the increased work of breathing necessary for survival, in addition to underlying comorbidity, can approach the nutritional gain from eating [2, 3]. As a result, many children with EOS are nutritionally depleted.

Children with EOS may be treated with various surgical techniques to improve their spine and/or chest wall deformity. One type of surgical treatment is the vertical expandable prosthetic titanium rib (VEPTR). The use of VEPTR in children with EOS has been shown to improve their weight [4]. Another technique to improve spine deformity in children with EOS is spine-based growing rod surgery. These growing rod constructs include pedicle screws, hooks, and wires fixed to the spine and rods that are periodically lengthened. Growing rods have been shown to improve Cobb angle, T1-S1 length, space available for the lung (SAL), and other radiographic parameters [5–7]. In addition, Bess et al. have reported on complications with growing rods. However, while radiographic outcomes and complications are certainly important, there is a paucity of literature examining the clinical outcomes and benefits of growing rod intervention in children with EOS [8].

The purpose of our study was to determine the nutritional status of children with EOS and to determine if treatment with growing rod surgery is associated with nutritional improvement.

## Materials and methods

A retrospectively collected multicenter EOS database of 284 patients was queried to identify those patients who underwent distraction-based growing rod surgery without rib-based implants and had a minimum follow-up of 24 months. We identified a subcohort of 88 patients who satisfied these criteria. The sample included patients from six different institutions.

Patient age at initial surgery, diagnosis, Cobb angle, weight, and length of follow-up were extracted from the database. Subjects were evaluated before surgery and after surgery, at varying intervals, until the final follow-up at least 24 months after initial surgery. Weight measurements and Cobb angles were recorded from each visit. All weights were converted to normative age-adjusted percentiles [9]. It is important to note that the normative data has a basement effect at ≤5th percentile. This means that a change in percentile from 2 percentile to 5 percentile would not be detected. Similarly, a change from 2nd percentile to 6th percentile would only show a change of 1 percentile. Thus, in children whose weights are ≤5th percentile before surgery, a subsequent gain in weight can be underestimated.

Paired Student *t* tests were used to compare preoperative and postoperative weight percentiles. Simple linear regression was used to examine the relationship between age at initial surgery and changes in weight percentile. Spearman rank order correlation coefficient procedures were used to measure the association between the change in weight percentile and preoperative Cobb angle. Pearson correlation coefficient procedures were used to measure the association between age at initial surgery and preoperative weight percentile, preoperative Cobb angle, or years of follow-up.

## Results

At the time of initial surgery, the mean age of the patients in our series was 5.8 years (range 1.4–11.5 years). All patients were observed for a minimum of 24 months, with a mean follow-up of 4.1 years (range 2–10 years). Diagnoses were categorized according to the classification system for EOS described by Williams et al. [10]. These include: idiopathic (23), congenital/structural (22), neuromuscular (22), and syndromic (21) scoliosis. Example etiologies of neuromuscular scoliosis include cerebral palsy, spinal muscular atrophy, myelodysplasia, and myopathy. Example etiologies of syndromic scoliosis include spondyloepiphyseal dysplasia, neurofibromatosis, and Marfan’s syndrome. The mean initial Cobb angle for the patients in our series was 75° (range 29°–145°). There was no significant relationship between preoperative Cobb angle and change in weight percentile (*p* = 0.52).

The mean preoperative weight in our study population was 18.1 kg (range 7.5–66.2 kg). The mean weight at final follow-up was 30.4 kg (range 12.3–82.5 kg). All of the patients gained weight at final follow-up. The mean absolute weight gain at final follow-up was 12.3 kg (range 0.3–41.3 kg).

Preoperatively, 47 % (41/88) of our patients were <5th percentile for weight, meeting the criteria for “failure to thrive.” Of these most nutritionally depleted patients, 44 % (18/41) showed increased weight percentiles at final follow-up. The mean improvement was 12 percentiles (range 1–69 percentiles). In the 23 patients who did not experience improvements in weight percentile, their percentile at final follow-up remained <5th percentile. However, all of these patients still gained absolute weight after surgery, but this was not sufficient to manifest as an increase in their weight percentile. Furthermore, the vast majority of these patients (20/23) were ≤1st percentile for weight at the time of their initial surgery. It is possible that a change in weight percentile may have occurred, but would not be recognized due to the basement effect of the ≤5th normative percentile. Of the 47 patients who were ≥5th percentile for weight before surgery, 51 % (24/47) showed an increase in weight percentile at final follow-up. Their mean improvement was 24 percentiles (range 3–70 percentiles).

**Fig. 1:**
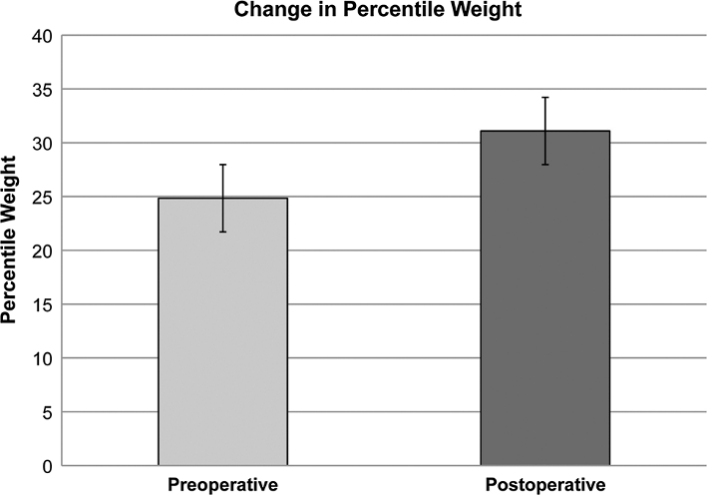
Change in weight percentile after treatment with growing rod surgery. There is a significant increase in the mean postoperative weight percentiles at latest follow-up (*p* = 0.004)

In total, 43 of 88 patients (49 %) gained weight percentile at final follow-up after growing rod surgery. Amongst those who gained weight percentile at final follow-up, the mean absolute weight gain was 14.5 kg, representing a mean gain of 18 percentiles. The mean improvement in weight percentile for our entire study population (88 patients) was statistically significant (*p* = 0.004) (Fig. 1).

A relationship exists between diagnosis and percentile weight gain. Children with neuromuscular and syndromic diagnoses do not appear to improve their mean nutritional status after surgery when compared to patients with idiopathic or congenital/structural scoliosis (*p* = 0.006). 22 of 88 patients (25 %) had neuromuscular diagnoses. These patients were followed for a mean of 3.6 years. Surprisingly, these patients were found to lose a mean of 1.3 weight percentiles during this period. Also, patients with syndromic diagnoses did not greatly improve their percentile weight gain. 21 of 88 patients (24 %) had syndromic diagnoses and gained only 3.1 weight percentiles during a mean follow-up of 4.7 years. On the other hand, children with idiopathic and congenital/structural diagnoses improved their mean percentile weight gain, 10.3 and 12. 5 weight percentiles, respectively. In these groups, only 31 % (7/22) and 33 % (7/21) of neuromuscular and syndromic scoliosis patients improved their percentile weight gain, respectively. In contrast, over half of the idiopathic and congenital/structural scoliosis patients improved the percentile weight gain (65 % (15/23) and 55 % (12/22), respectively). This relationship was not confounded by preoperative weight percentile (*p* = 0.59), preoperative Cobb angle (*p* = 0.59), or years of follow-up (*p* = 0.72).

**Fig. 2:**
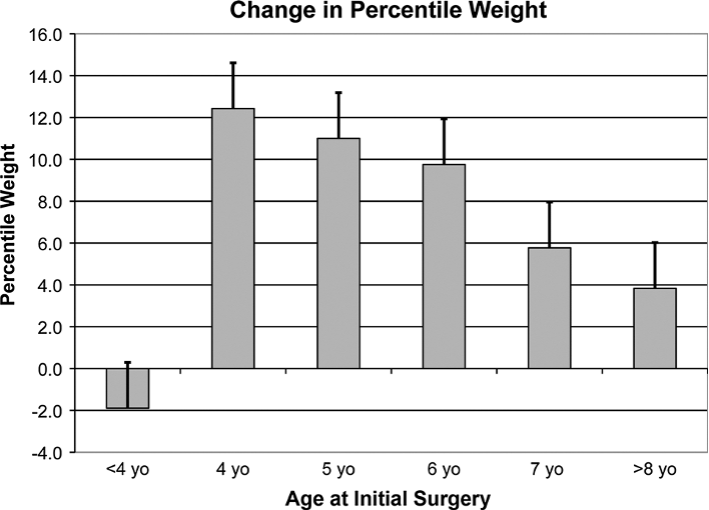
Change in weight percentile as a function of age at initial growing rod surgery. Children <4 years old do not appear to improve their mean weight percentile after surgery. Improvement in weight percentile was greatest at age 4 and decreased in a linear fashion with increasing age thereafter

A significant relationship exists between age at initial surgery and percentile weight gain (Fig. 2). Children <4 years old do not appear to improve their mean nutritional status after surgery (*p* < 0.005). Prior to surgery, 19 of 88 patients (22 %) were <4 years old. These patients were followed for a mean of 3.9 years. Surprisingly, these patients were found to lose a mean of 1.9 weight percentiles during this period. On the other hand, children of age 4 years gained a mean of 13.4 kg, representing a mean gain of 12.4 weight percentiles. Improvement in nutritional status was greatest at age 4 and decreased in a linear fashion with increasing age thereafter (*p* = 0.004). This relationship was not confounded by preoperative weight percentile (*p* = 0.25), preoperative Cobb angle (*p* = 0.095), or years of follow-up (*p* = 0.95). Patients who were initially ≤5th percentile for weight were evenly distributed across all age groups.

While there is no difference in preoperative Cobb angles between children <4 years old and children >4 years old, there are differences in their underlying diagnoses. These differences, however, are not significant. In children <4 years old, a greater percentage of patients have neuromuscular diagnoses (7/19, 37 %) as compared to children >4 years old (15/69, 22 %) at initial surgical intervention (*p* = −0.30). On the other hand, in children <4 years old, a lesser percentage of patients have syndromic diagnoses (2/19, 11 %) as compared to children >4 years old (19/69, 28 %) (*p* = 0.22). There were similar frequencies of idiopathic and congenital/structural diagnoses between the two groups. In children <4 years old, 5 patients (26 %) had idiopathic scoliosis, and 5 patients (26 %) had congenital/structural scoliosis. In children ≥4 years old, 18 patients (26 %) had idiopathic scoliosis, and 17 patients (25 %) had congenital/structural scoliosis.

## Discussion

According to the Center for Disease Control Pediatric and Pregnancy Nutrition Surveillance System, underweight is defined as weight-for-age <5th percentile and body mass index (BMI)-for-age <5th percentile for children 2–20 years of age based on the CDC gender-specific BMI-for-age reference [9]. For children with EOS, BMI is not a reliable measure of nutrition, as it relies on the height measurement. For our purposes, the term “failure to thrive” has also been applied to children <5th percentile for body weight [11].

In our study, there was significant improvement in nutritional status in approximately 50 % of patients following treatment of EOS with growing rods, similar to that reported with VEPTR [4]. In those patients initially considered to have “failure to thrive” (≤5th percentile), 44 % showed an increase in weight percentile after surgery, also similar to that reported with VEPTR. In these children with “failure to thrive,” this increase is likely an underestimate due to the basement effect of normative percentiles in this range.

It is difficult to determine exactly what amount of percentile weight gain is clinically relevant with regards to outcomes such as infection, neurodevelopment, post-operative complications and mortality. Several studies show that maintaining patients out of the malnourished range and at least maintaining their age-appropriate weight decreases risk of infectious complications during hospitalization, post-operative length of stay, unplanned readmissions, and mortality during the course of their treatments [12–15]. One study in the cardiac literature showed that, in children with congenital heart disease who were matched for complexity of congenital heart surgical procedures, the odds ratio for death was 13.5 if there was a decrease in the weight-for-age *Z*-score of at least 0.67 after the last operation. Certainly, this is a significantly different patient population than our in our study, but it brings attention to the concern regarding weight loss in fragile children [13].

The relationship between age at initial surgery and nutritional improvement is intriguing. Our data demonstrates that children <4 years old at the time of surgery do not improve their nutritional status even up to 4 years after surgery. In addition, these children under age 4 years more often had underlying neuromuscular diagnoses. This difference in diagnoses may account for the lack of gain in weight percentile, as it may reflect deficiencies that began in utero and are not yet stabilized in early childhood. Several studies have found an increased rate of malnutrition in developmentally disabled patients, as this can be attributed to problems feeding and increased time and assistance from caretakers [16, 17]. Recently, Bess et al. [8] found a 13 % decrease in complications with each additional year of age at initial surgery, concluding that there are fewer complications when surgery is performed at a later age. This same study also demonstrated a 24 % increase in complications with each additional procedure. More recently, Sankar et al. [18] described the “law of diminishing returns,” wherein the gain in T1-S1 length tends to decrease with each subsequent lengthening over time. Our current study, in combination with the study by Bess et al. and the “law of diminishing returns,” reports benefits of delaying the initial surgery. When considering timing of initial surgical intervention, attention to young age and underlying diagnosis, such as neuromuscular or syndromic diagnosis, are both important risk factors for potentially less favorable nutritional status in the course of treatments. Further peri-operative optimization or attention may be needed in these patients.

Also, the efficacy of non-operative modalities, such as casting or bracing, in an effort to delay initial surgical intervention should be further explored. Delaying surgery must, of course, be weighed against the best growth potential and the harm of worsening untreated spinal deformity. To date, we are not aware of any studies examining the effect of derotational casting in children with EOS on nutritional status. However, recent studies have shown that derotational casting may effectively delay surgical intervention in patients with moderate-to-severe EOS, on average 39 months [19]. This study also references the same concerns regarding the timing of surgical intervention and complication rates in children with EOS. Also, nearly half of the patients in this study were <5 years old, which includes the age group of patients in our study that did not improve their nutritional status. However, clinical outcomes in the casted patients with respect to nutritional status were not examined. Also, 41 % (12/29) of patients in the casting study have idiopathic scoliosis, and 6.9 % (2/29) of patients have neuromuscular scoliosis. Sanders et al. [20] also demonstrated the efficacy of derotational casting with respect to radiographic parameters in idiopathic scoliosis patients whose mean age was 2.2 years at first cast. Recently, Dhawale et al. [21] examined the clinical outcome of peak inspiratory pressures (PIPs) during the course of casting in very young patients with idiopathic and syndromic EOS. Interestingly, PIPs did not return to baseline after cast windows were cut out. As such, the authors urge caution during the casting process in patients with underlying pulmonary disease. Certainly, derotational casting plays an important role in our armentarium of treatment for EOS. Further studies regarding clinical outcomes are necessary and will be interesting.

An inherent limitation of this case series is the lack of a control cohort. While this study provides comparisons of growing rod surgery to VEPTR, we currently lack data regarding the natural history of nutritional status in children with EOS who are not treated with surgical intervention for spine deformity, as withholding surgical care is often not a desirable option in these severely ill patients. It is also possible that the close attention given to these children during the postoperative period could result in a better diet and weight gain independent of growing rod implantation. Improvements in weight, however, appear to persist even 48 months after surgery, which is long after the acute postoperative period during which the child would receive the most attention. In addition, all centers routinely maximized patients’ nutritional status prior to the initial surgery. A further limitation of the study is that we lack data regarding the presence or absence of gastrostomy tube intervention or major hospitalizations for infection and other illnesses, and we do not have information on the quality of nutritional support given to the patients post-operatively. As these children are very fragile, a single extensive hospitalization for infection can result in significant weight losses. Furthermore, children with EOS often undergo additional procedures during the course of their spinal deformity treatment such as tracheostomy placement or decannulation, baclofen pump placement, and cardiac procedures that may significantly affect overall nutritional status. These variables warrant further review in future prospective studies. However, from this current study, we strive to improve future prospective database collection and bring attention to the question of timing with respect to growing rod treatment initiation and to the examination of clinical outcomes.

This study highlights the very poor nutritional status of children with EOS, with nearly half of the patients being <5th percentile in weight prior to surgery. Our data also support an improvement in weight percentile of children with EOS after growing rod surgery, which is a critically important outcome measure in this fragile population. This study also shows that patients with underlying diagnoses of neuromuscular scoliosis do not gain weight percentile after surgical intervention. Finally, this data shows that children < 4 years old at the time of initial surgery do not gain weight percentile after surgery; however, weight percentile gain is greatest when initial surgery is performed at age 4 years and linearly decreases thereafter. These data have important implications with regards to the timing of initial surgical intervention in children with EOS with respect to age and diagnosis. The treatment of EOS in very young children with neuromuscular diagnoses may present an even more difficult and unique challenge in the study of EOS.
